# Bidirectional Mechanisms Linking Circadian Rhythm Disruption and Parkinson’s Disease: Chronobiomarkers and Therapeutic Implications

**DOI:** 10.3390/ijms27156719

**Published:** 2026-07-28

**Authors:** Xinyue Zhang, Weina Shen, You Wu, Wei Zhang, Qing Ye

**Affiliations:** Longhua Hospital, Shanghai University of Traditional Chinese Medicine, Shanghai 200032, China; xyzhang@shutcm.edu.cn (X.Z.); weinashen@aliyun.com (W.S.); kathyyouyou@126.com (Y.W.); 12024116@shutcm.edu.cn (W.Z.)

**Keywords:** Parkinson’s disease, circadian rhythm disruption, α-synuclein, clock genes, glymphatic system, gut microbiota

## Abstract

Parkinson’s disease (PD) is a progressive neurodegenerative disorder in which circadian rhythm disruption (CRD) emerges as both a prodromal feature and a potential pathogenic driver. Elucidating the bidirectional interplay between PD and CRD is essential for identifying early biomarkers and developing chronotherapeutic strategies. We narratively synthesized literature published over the past two decades in PubMed, Web of Science, and CNKI, focusing on molecular mechanisms, clinical manifestations, biomarker development, and interventional studies addressing the PD–CRD interface. In the CRD-PD direction, circadian disruption accelerates dopaminergic neurodegeneration through four convergent mechanisms: (i) REV-ERBα–mediated dysregulation of dopamine biosynthesis and NF-κB/NLRP3-driven neuroinflammation; (ii) impaired sleep-dependent glymphatic clearance of α-synuclein (α-syn); (iii) NAD^+^–SIRT1–BMAL1–PGC-1α axis dysfunction leading to mitochondrial bioenergetic failure; and (iv) C/EBPβ-dependent autophagic rhythm disruption coupled with pro-inflammatory microglial activation, collectively establishing a dual pro-inflammatory–autophagy-suppressive milieu permissive for α-syn aggregation. In the reverse PD-CRD direction, PD pathology destabilizes the circadian system via Braak-stage degeneration of rhythm-regulatory nuclei, retinal dopaminergic denervation attenuating SCN photic entrainment, pineal–melatonin axis suppression, iatrogenic effects of dopaminergic pharmacotherapy, and gut microbiota dysbiosis propagated through the microbiota–gut–brain axis. Emerging multi-modal chronobiomarkers—including peripheral clock gene expression profiles, melatonin secretion patterns, tryptophan–kynurenine metabolites, and gut microbial oscillation signatures—show promise for prodromal diagnosis and disease subtyping. Circadian-targeted precision interventions—encompassing timed bright light therapy, exogenous melatonin, and chronopharmacological interventions—represent a promising translational paradigm for the early identification and management of PD.

## 1. Introduction

Parkinson’s disease (PD) is a neurodegenerative disorder pathologically characterized by progressive loss of dopaminergic neurons in the substantia nigra pars compacta (SNpc) of the midbrain and the formation of Lewy bodies composed of α-synuclein (α-syn) [1]. In addition to the classical motor symptoms, sleep disorders and circadian rhythm disruption among the non-motor symptoms (NMS) are widely present in the prodromal stage of PD and significantly impair patients’ quality of life [2,3]. Contemporary conceptualizations of Parkinson’s disease have undergone a paradigm shift from a purely motor disorder to a systemic, multi-dimensional disease entity in which non-motor symptoms (NMS) constitute core diagnostic and prognostic dimensions rather than secondary manifestations. The evolving NMS spectrum—encompassing circadian rhythm disruption, rapid eye movement sleep behavior disorder (RBD), olfactory dysfunction, autonomic dysregulation, and neuropsychiatric features—often precedes the emergence of cardinal motor symptoms by years to decades, providing a critical window for early disease identification and neuroprotective intervention. The temporal sequence of prodromal NMS, anchored by isolated RBD as the strongest conversion predictor (>80% phenoconversion to α-synucleinopathy within 10–15 years), has been further refined by the integration of chronobiological biomarkers (blunted melatonin rhythmicity, attenuated circadian amplitude) and gut microbiota dysbiosis signatures, collectively informing a precision medicine framework for individualized risk stratification and chronotherapeutic management.

Mammalian circadian rhythms are governed by the suprachiasmatic nucleus (SCN) of the hypothalamus as the master pacemaker, which maintains an oscillation period of approximately 24 h through a transcription–translation feedback loop (TTFL) composed of core clock genes including *CLOCK*, *BMAL1*, *PER1/2*, and *CRY1/2* [4]. This system regulates a variety of physiological processes such as sleep–wake cycles, hormonal secretion, body temperature, metabolism, and immunity.

Recent studies have revealed a bidirectional relationship between PD and CRD: on one hand, degeneration of the rhythm system accelerates the progression of PD pathology through mechanisms such as imbalanced dopamine biosynthesis, impaired α-syn clearance, oxidative stress, and neuroinflammation [5,6]; on the other hand, PD pathology itself disrupts the rhythm system by impairing the SCN and related neural pathways [7,8]. This review focuses on the bidirectional mechanisms and rhythm-targeted clinical diagnostic and therapeutic strategies. Whereas previous reviews have predominantly addressed the unidirectional impact of CRD on PD, this review synthesizes recent evidence supporting a bidirectional feedback-loop model linking CRD and PD and highlights emerging chronobiomarkers and chronotherapeutic strategies.

Although several reviews published in the past three years have addressed the interplay between CRD and PD, most of them remain confined to a parallel description of individual molecular pathways or clinical phenomena. A systematic delineation of the hierarchical relationships between upstream driving mechanisms and downstream effector mechanisms is still lacking, and the integration of emerging peripheral biomarker evidence remains incomplete. Against this background, the present review provides substantive additions to the existing literature in the following two aspects:

(1) Systematic organization of the hierarchical relationships among four converging pathogenic mechanisms. Unlike previous reviews that merely enumerate discrete pathways—such as *REV-ERBα*-mediated neuroinflammation, impairment of glymphatic clearance, NAD^+^–SIRT1-related mitochondrial dysfunction, and disruption of autophagic rhythms—this review systematically situates these four mechanisms within a unified causal framework. Specifically, dysregulation of the core clock-gene transcriptional network is positioned as the upstream initiating node; microglial NF-κB/NLRP3 activation and C/EBPβ-dependent autophagy suppression are defined as the intermediate amplifying nodes; and impaired α-syn clearance together with dopaminergic neurodegeneration serves as the downstream terminal effector node. This hierarchical mapping clarifies the temporal sequence, cross-talk, and potential therapeutic priority among these mechanisms, thereby providing a mechanistic rationale for the future selection of single-target or multi-target combinatorial interventions.

(2) Inclusion of gut-microbiota rhythmicity markers in a multimodal chronobiomarker panel. Although gut dysbiosis has been extensively reported in the prodromal phase of PD, existing chronobiomarker frameworks have focused primarily on peripheral clock-gene expression, melatonin/cortisol secretion rhythms, and the tryptophan–kynurenine metabolic pathway, without systematically incorporating microbiota-related rhythmic indicators. Building on the current biomarker system, this review newly integrates several dimensions—including 24 h oscillation patterns of fecal microbial communities, diurnal fluctuations in the abundance of short-chain fatty acid (SCFA)-producing taxa, and rhythmic metabolic signatures of bile acids and tryptophan derivatives. Coupled with timed sampling, host feeding-rhythm normalization, and adjustment for pharmacological confounders, these additions constitute a multimodal chronobiomarker framework that integrates central, peripheral, and gut-level dimensions, offering a novel translational pathway for prodromal screening, subtype stratification, and treatment monitoring in PD.

## 2. Literature Search Strategy and Evidence Grading Framework

This review was conducted as a narrative synthesis of the literature. A comprehensive literature search was performed in PubMed, Web of Science, and CNKI (China National Knowledge Infrastructure), covering publications from 2003 to June 2026, with emphasis on studies published after 2020. The search strategy employed the following keyword combinations: (“Parkinson” OR “PD”) AND (“circadian rhythm” OR “circadian disruption” OR “clock gene” OR “melatonin” OR “sleep” OR “glymphatic” OR “REV-ERB” OR “SIRT1” OR “autophagy” OR “gut microbiota” OR “chronotherapy” OR “chronobiomarker”). Additional relevant articles were identified through citation tracking and manual screening of reference lists from key reviews.

Inclusion criteria comprised: (i) original research articles and authoritative reviews addressing the bidirectional relationship between CRD and PD; (ii) studies reporting molecular mechanisms, clinical manifestations, biomarker validation, or interventional outcomes at the PD-CRD interface; (iii) human studies (cohort, case-control, cross-sectional, randomized controlled trials), animal models (MPTP, 6-OHDA, α-syn preformed fibrils, genetic models), and cellular experiments. Exclusion criteria included: conference abstracts, non-peer-reviewed sources, editorials without primary data, and studies not directly addressing circadian rhythm or clock gene function in the context of PD. No language restriction was applied for CNKI searches of Chinese-language literature.

To critically appraise the translational relevance of cited evidence, this review adopts E/P/H evidence grading system applied throughout the manuscript and in all summary tables:

Established (E): Findings supported by multiple independent human studies (cohort, case-control, or randomized controlled trials) with convergent mechanistic evidence from direct pathway manipulation in disease-relevant models.

Probable (P): Mechanistic findings robustly demonstrated in animal and/or in vitro models with preliminary human observational evidence; causal pathway manipulation shows consistent effects across independent studies but human interventional data remain limited.

Hypothetical (H): Evidence derived primarily from non-PD model systems or based on correlative observations without direct pathway manipulation in PD-relevant contexts; extrapolation to human PD pathobiology requires cautious interpretation.

**Figure 1:**
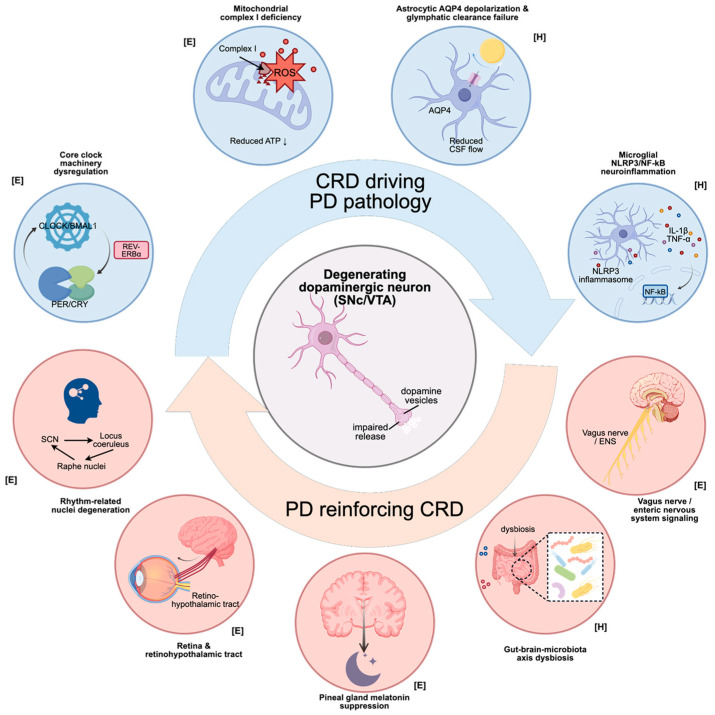
The Bidirectional Vicious Cycle Between CRD and PD. Upper arc: Dysregulation of the core circadian clock transcriptional network—*CLOCK/BMAL1*, *PER/CRY*, *REV-ERBα*/*RORα*—triggers four pathogenic cascades: mitochondrial complex I deficiency and ROS overproduction; AQP4 depolarization leading to glymphatic clearance failure; microglial NLRP3/NF-κB–mediated neuroinflammatory activation. Collectively, these cascades drive progressive degeneration of SNc/VTA dopaminergic neurons. Lower arc: Degeneration of rhythm-related nuclei—including the SCN, locus coeruleus, and raphe nuclei—impairs retinal photic input to the SCN via the RHT, suppresses pineal melatonin secretion, disrupts gut–brain–microbiota axis homeostasis, and perturbs vagus nerve/ENS signaling.

Each entry in Table 1, Table 2 and Table 3 and Figure 1 is annotated with the corresponding evidence level (E/P/H) and the model or sample type from which the finding was derived, enabling readers to distinguish established clinical findings from preclinical or hypothetical mechanisms. Greater interpretive weight is assigned to studies with direct pathway manipulation, appropriate controls, and functional readouts in human-relevant systems.

## 3. Clinical Manifestations of CRD in PD

### 3.1. Disruption of the Sleep–Wake Cycle

RBD is one of the strongest prodromal biomarkers of PD. Isolated RBD (iRBD) phenoconverts to α-synucleinopathies (including PD and dementia with Lewy bodies, DLB) at rates of ~73.5% at 12 years and >80% at 14 years [9,10]. The prevalence of insomnia and excessive daytime sleepiness (EDS) in PD patients reaches 30–80% and 15–50%, respectively [11]. The incidence of restless legs syndrome (RLS) in PD patients is also significantly higher than that in the general population and is closely related to dopaminergic dysfunction [12].

**Table 1:** Clinical Summary of Circadian and Sleep Manifestations in PD: Prevalence, Assessment, Mechanism, and Clinical Significance [13,14].

Symptom	Estimated Prevalence in PD	Objective Assessment Tool	Mechanism	Clinical Significance
RBD [15]	>70% (prodromal; 80% conversion in 10–15 yr)	Video-PSG; ICSD-3 criteria	Brainstem (LC, PPN) α-syn deposition; clock gene dysregulation (*BMAL1*, *PER2*)	Strongest prodromal marker; predicts faster cognitive decline
Insomnia	30–80%	PDSS-2; SCOPA-SLEEP; ISI [16]	SCN output attenuation; melatonin phase advance; nocturnal motor symptoms [17]	Impairs QoL; exacerbates daytime fatigue and cognitive dysfunction
EDS/Sleep attacks [18]	15–50%	ESS; MDS-NMS; MSLT	Orexinergic neuron loss; dopaminergic medication sedation; SCN dysregulation	Safety hazard; associated with cognitive impairment and falls
RLS [19]	5–20%	IRLS scale; suggested clinical criteria	Dopaminergic dysfunction; iron metabolism circadian disruption	Disrupts sleep onset; may mimic or aggravate akathisia
Nocturia	~60%	Bladder diary; NMSS	Loss of ADH circadian rhythm; autonomic dysfunction; detrusor overactivity	Sleep fragmentation; fall risk during nocturnal toileting
Sundowning	Variable (PD-MCI/PDD)	Neuropsychiatric inventory	Cholinergic circadian dysregulation; SCN degeneration; insufficient light exposure	Predicts dementia progression; caregiver burden

### 3.2. Disruption of Core Physiological Rhythms

Beyond the sleep–wake cycle, multiple endogenous circadian physiological rhythms are characterized by attenuated amplitude, phase shift, or rhythm abolition in PD patients.

#### 3.2.1. Abnormal Melatonin Secretion Rhythm

In healthy individuals, plasma melatonin exhibits a typical nocturnal peak. In PD patients, the nocturnal melatonin peak is reduced in amplitude and phase-delayed; the secretion curve becomes flattened, with rhythm disturbance being more pronounced in those receiving long-term levodopa therapy (disease effect and medication effect are separable but compounding) [20]. This change is associated with reduced pineal function and attenuated noradrenergic drive from the SCN to the pineal gland, constituting a key biochemical basis for insomnia and circadian phase disturbances.

#### 3.2.2. Core Body Temperature and Cortisol Rhythms

In PD patients, core body temperature circadian amplitude is reduced with advanced nocturnal nadir, and cortisol rhythm displays a flattened pattern with attenuated morning peak, indicating HPA axis dysregulation. These neuroendocrine disturbances are reciprocally associated with depression and cognitive impairment [21].

#### 3.2.3. Inversion of Blood Pressure and Autonomic Rhythms

A normal circadian blood pressure pattern is “dipping”, with a 10–20% nocturnal decline. In PD patients, however, “non-dipping” or “reverse-dipping” patterns occur in over 50%, often combined with orthostatic hypotension and postprandial hypotension, leading to nocturnal supine hypertension and morning orthostatic hypotension, which significantly increases cardiocerebrovascular risk [8]. Attenuated circadian rhythm of heart rate variability may also serve as an early marker of autonomic dysfunction [22].

### 3.3. Circadian Fluctuation of Motor Symptoms

The motor symptoms of PD themselves exhibit prominent circadian variation: most patients respond well in the morning during the “on” period, then experience worsening end-of-dose phenomena from afternoon to evening, while nocturnal rigidity, tremor, and difficulty turning in bed seriously impair sleep. These fluctuations stem from circadian differences in levodopa pharmacokinetics, circadian variation in striatal dopamine receptor sensitivity, and SCN regulation of motor cortex projections (tentative mechanism, based on limited preclinical evidence). Accurate clinical identification requires continuous 24 h recording via “motor diaries” or wearable motion sensors.

### 3.4. Circadian Features of Non-Motor Symptoms

#### 3.4.1. Cognitive Fluctuation

PD patients, particularly those with cognitive impairment (PD-MCI, PDD), commonly experience “sundowning”: decreased attention, disorientation, agitation, or hallucinations from evening to night. The mechanisms involve abnormal circadian regulation of the cholinergic system, SCN degeneration, and insufficient light exposure [23].

#### 3.4.2. Mood and Psychiatric Symptoms

Depression and anxiety in PD exhibit either morning-worse–evening-better or reverse fluctuation patterns, related to abnormal circadian release of monoaminergic neurotransmitters (e.g., 5-HT, NE) [24]. Dysregulated circadian control of the “tryptophan–kynurenine” metabolic pathway may serve as a common mechanism linking CRD with mood disorders and neurodegeneration in PD. Visual hallucinations are more frequent at dusk and at night, suggesting circadian instability in the visual cortex–thalamus–brainstem circuitry.

#### 3.4.3. Gastrointestinal Function and Gut Microbiota Rhythm

Constipation is one of the earliest non-motor symptoms of PD, closely related to enteric α-syn deposition and circadian disruption of gut microbiota. Studies have shown that the circadian oscillation amplitude of gut microbiota in PD patients is reduced, with abolition of the rhythmicity of short-chain fatty acid (SCFA)-producing bacteria, potentially feeding back onto central SCN function via the “microbiota–gut–brain axis” [25]. These findings suggest potential—but currently unvalidated—avenues for FMT and chronotherapeutic probiotic interventions, which require rigorous human interventional trials before clinical application.

#### 3.4.4. Urination and Metabolic Rhythms

Nocturia is observed in approximately 60% of PD patients, attributable to the loss of nocturnal antidiuretic hormone secretion rhythm, detrusor overactivity, and autonomic dysfunction. Regarding glucose metabolism, the circadian rhythm of insulin sensitivity is also impaired, and some patients exhibit “morning hyperglycemia–nocturnal hypoglycemia” fluctuations, complicating the management of comorbid diabetes.

## 4. Bidirectional Mechanisms Linking Circadian Disruption and PD Neurodegeneration

The relationship between PD and the circadian rhythm system is not a simple linear symptom–disease association but constitutes a self-amplifying vicious feedback loop. On one hand, exogenous or endogenous CRD accelerates the degeneration of nigral DA neurons through multiple molecular pathways; on the other hand, the core pathological processes of PD—abnormal α-syn aggregation, caudo-rostral spread of Lewy pathology, and loss of dopaminergic input—reciprocally destabilize central and peripheral biological clocks.

### 4.1. Rhythm System Degeneration Drives or Accelerates PD Pathology

#### 4.1.1. Circadian Transcriptional Network Regulation of Dopamine Biosynthesis

Dopamine synthesis in the VTA and SNpc exhibits prominent circadian rhythmicity, with the rate-limiting enzyme tyrosine hydroxylase (TH) being precisely regulated by the core clock gene network. As a member of the nuclear receptor superfamily, *REV-ERBα* recognizes the *REV-ERB* response elements (RevRE/RORE; core motif AGGTCA half-site) within the TH promoter, competitively inhibits RORα-mediated transcriptional activation, thereby achieving circadian negative regulation of TH expression, and—through antagonism with the orphan nuclear receptor NURR1 (NR4A2)—performs rhythmic “on/off” regulation of midbrain dopaminergic neuronal identity and DA biosynthesis [26]. In Rev-erbα-deficient mouse models, the circadian amplitude of DA synthesis is significantly abolished, accompanied by late-onset midbrain neuronal degeneration, suggesting that clock gene dysregulation per se exhibits a neurodegenerative phenotype [27]. Moreover, conditional knockout of *BMAL1* in mice results in age-dependent oxidative stress damage, astrocytic activation, and synaptic degeneration [28]. Beyond the originally described redox homeostasis disruption, emerging evidence indicates that circadian clock protein dysfunction simultaneously engages multiple convergent pathogenic cascades—including NLRP3 inflammasome-mediated neuroinflammation, chaperone-mediated autophagy suppression, and mitochondrial bioenergetic failure—thereby establishing a multi-dimensional pathological network rather than a unidimensional oxidative stress mechanism. Disease-specific alterations in clock gene expression profiles, particularly the abolition of *REV-ERBα* circadian oscillation and aberrant *PER1/PER2* promoter methylation identified in PD patients, may serve as early-stage molecular indicators of circadian disruption preceding overt motor manifestations.

In the MPTP model, SR9009 (a selective *REV-ERBα* agonist) suppresses microglial NLRP3 inflammasome activation, promotes M1→M2 polarization, and partially rescues nigrostriatal dopaminergic loss and motor deficits [29]. Conversely, Nr1d1^−^/^−^ mice show upregulated astrocytic C3/C4b and enhanced complement-dependent synaptic phagocytosis, positioning *REV-ERBα* as a multilayered neuroprotective node [E].

#### 4.1.2. Sleep-Dependent Glymphatic Clearance and α-Syn Pathology

Hablitz et al. demonstrated that glymphatic influx and clearance exhibit endogenous circadian rhythms peaking during the mid-rest phase, with daytime influx approximately 22% higher than nighttime levels [30]. This rhythm persists under constant darkness conditions, confirming its endogenous circadian origin rather than a mere light-dark masking response. Critically, the perivascular polarization of AQP4—rather than its total expression level—exhibits robust circadian oscillation, driven by the rhythmic expression of the dystrophin-associated protein complex (DAC) components including Dag1, Dtna, and Gja1 at astrocytic endfeet [31]. Genetic ablation of Aqp4 abolishes both the day-night difference in glymphatic influx and the circadian variation in cervical lymph node drainage, establishing AQP4 polarization as the necessary molecular link between the core clock and CSF distribution dynamics. Notably, CSF distribution follows a bidirectional anti-phase rhythm: glymphatic influx into the brain parenchyma peaks during the rest phase, whereas drainage to peripheral lymph nodes peaks during the active phase—suggesting a time-of-day-dependent switching mechanism governing CSF allocation between intra- and extra-parenchymal compartments [32]. Diffusion tensor imaging analysis along the perivascular space (DTI-ALPS index) has likewise revealed significantly impaired glymphatic function in PD patients, correlating with motor symptom severity and α-syn burden [10]. Sleep deprivation elevates cerebrospinal fluid α-syn and tau concentrations [33], indicating that disruption of rhythmic sleep architecture directly weakens the nocturnal clearance of pathogenic proteins, accelerating the formation of Lewy pathology.

SCN, serving as the master circadian pacemaker in mammals, exhibits a rhythmic oscillation in spontaneous neuronal firing rate characterized by elevated daytime and reduced nighttime activity. The amplitude of this electrophysiological oscillation constitutes a core determinant of the robustness of the circadian timekeeping system. Ambient light intensity modulates circadian system stability through its regulatory effects on SCN electrophysiological amplitude: under high-irradiance conditions, the peak daytime firing rate of SCN neurons is markedly augmented, with the resting membrane potential (RMP) shifting toward a more depolarized state; conversely, under low-irradiance conditions, a substantial proportion of SCN neurons adopt a hyperpolarized quiescent state during the subjective day, resulting in a pronounced attenuation of circadian amplitude [34]. Notably, this effect persists under constant darkness free-running conditions following the withdrawal of photic stimulation, indicating that it does not represent an acute masking response driven by immediate photic input, but rather reflects a long-lasting remodeling of the intrinsic physiological properties of the SCN neuronal network. Within the pathological context of PD, the decline in retinal dopamine levels—resulting from the progressive degeneration of dopaminergic amacrine cells—leads to an attenuation of photic signal transduction along the retinohypothalamic tract (RHT), which may in turn diminish SCN oscillatory amplitude and thereby compromise the resilience of the entire circadian system against exogenous and endogenous perturbations, further accelerating the degenerative cascade of nigrostriatal dopaminergic neurons.

#### 4.1.3. The SIRT1–BMAL1–PGC-1α Axis and Mitochondrial Dysfunction

A profound bidirectional coupling exists between the circadian timekeeping system and cellular energy metabolism, with the NAD^+^–SIRT1–BMAL1 positive feedback loop [35] serving as the central integrative nexus. The CLOCK:BMAL1 heterodimer drives transcription of nicotinamide phosphoribosyl transferase (Nampt), whose rhythmic expression ensures robust circadian oscillations in intracellular NAD^+^ concentrations [36]. NAD^+^-dependent SIRT1 deacetylates BMAL1 and PER2, stabilizing the core transcription–translation feedback loop and establishing a self-reinforcing circuit (CLOCK:BMAL1 → NAMPT → NAD^+^ → SIRT1 → BMAL1) [37]. Beyond this loop, SIRT1 activates PGC-1α to orchestrate mitochondrial biogenesis, while SIRT3 and SIRT6 cooperate in partitioning the circadian epigenome across mitochondrial and nuclear compartments.

Within the pathological context of PD, aging and circadian disruption synergistically drive a progressive decline in NAD^+^ bioavailability and attenuation of SIRT1 enzymatic activity [38], leading to the pathological accumulation of hyperacetylated BMAL1, diminished mitochondrial electron transport chain complex I activity, and the intraneuronal accumulation of DAQ and ROS within DA neurons—a pathophysiological signature that consistent with the mitochondrial complex I deficiency reported in idiopathic PD by Flønes et al. [39,40]. This mechanistic framework is further corroborated at the genetic level: in PTEN-induced kinase 1 (PINK1) loss-of-function mutant models, the NAD^+^ salvage biosynthesis pathway is significantly compromised. Notably, supplementation with NAD^+^ precursors—such as nicotinamide mononucleotide (NMN) and nicotinamide riboside (NR) [41]—or pharmacological activation of SIRT1 has been shown to restore circadian oscillatory amplitude and confer neuroprotective effects on dopaminergic neurons across multiple experimental PD models [42], highlighting the translational therapeutic potential of targeting the NAD^+^–SIRT1–BMAL1 axis as a disease-modifying intervention strategy in PD.

#### 4.1.4. CRD-Mediated Neuroinflammation and Autophagy Deficits

Clock genes are expressed not only in neurons but also widely in microglia. Loss of Bmal1 or *REV-ERBα* significantly upregulates microglial TLR4/NF-κB signaling, promoting the release of proinflammatory cytokines such as IL-6 and TNF-α. Griffin et al. were the first to demonstrate that *REV-ERBα* knockout mice exhibit spontaneous hippocampal inflammation and synaptic loss [43], indicating its protective role in neurodegeneration. Concurrently, autophagy-related genes (LC3B, ULK1, TFEB) are rhythmically driven by BMAL1; rhythm disruption attenuates chaperone-mediated autophagy (CMA), which is a major pathway for α-syn degradation. Therefore, CRD provides a permissive environment for α-syn aggregation through the dual mechanism of pro-inflammation–autophagy suppression.

The following mechanistic evidence is primarily derived from mouse hepatic models and Drosophila neurodegeneration models. Extrapolation to human midbrain dopaminergic neurons requires cautious interpretation, as tissue-specific autophagy regulation may differ substantially between hepatic and neuronal systems.

The circadian timekeeping system orchestrates cellular clearance mechanisms. Ma et al. demonstrated that hepatic autophagic activity in mice exhibits robust circadian rhythm, peaking at Zeitgeber time 6–9 (ZT6–9) and declining during the dark phase [44]. This rhythmic activation is accompanied by coordinated expression of autophagy-related genes (Ulk1, Gabarapl1, LC3B, Bnip3, Atp6v1d) [45]. CCAAT/enhancer-binding protein beta (C/EBPβ) serves as the pivotal transcription factor: driven by the hepatic molecular clock with peak expression at ZT13, it directly activates autophagy effector genes (Gabarapl1, Bnip3, Ctsl). In hepatocyte-specific Bmal1 knockout mice, C/EBPβ oscillation was markedly attenuated and autophagy gene rhythmicity virtually abolished, establishing tissue-autonomous regulation of autophagic rhythmicity by the core clock.

The aforementioned findings bear direct mechanistic implications for PD pathobiology. The proteolytic degradation of α-syn is critically dependent upon both the chaperone-mediated autophagy (CMA) pathway and the macroautophagy pathway, both of which are subject to temporal gating by the circadian system. Circadian disruption, by attenuating C/EBPβ-driven transcriptional activation of the autophagic gene program, compromises lysosomal degradative efficiency and thereby creates a permissive intracellular milieu conducive to the pathological accumulation and aggregation of α-syn. In Drosophila neurodegeneration models, loss of period (per^01^) synergizes with oxidative stress mutations (sni^1^, sws), reducing lifespan by 32–50% and exacerbating neuropathology through pathways independent of oxidative stress alone [46]. However, extrapolation from hepatic autophagy models and invertebrate systems to human midbrain dopaminergic neurons requires cautious interpretation.

#### 4.1.5. Integration and Hierarchy of the Four Mechanisms

**Figure 2:**
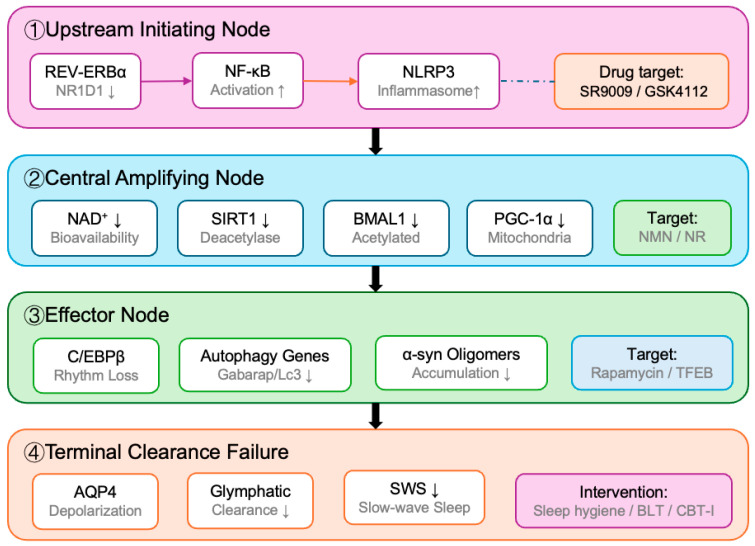
Four-Tier Hierarchical Model of the REV-ERBα/NLRP3 Axis in CRD–PD Pathogenesis. The downward arrow (↓) indicates gene downregulation or decreased production. Tier 1 (Upstream Initiating): REV-ERBα (NR1D1) loss → NF-κB activation → NLRP3 inflammasome upregulation; targetable by SR9009/GSK4112. Tier 2 (Central Amplifying): NAD^+^ ↓ → SIRT1 ↓ → hyperacetylated BMAL1 + suppressed PGC-1α → mitochondrial dysfunction; targetable by NMN/NR. Tier 3 (Effector): C/EBPβ rhythm loss → autophagy genes (Gabarap, Lc3) ↓ → α-synuclein oligomer accumulation; targetable by Rapamycin/TFEB activation. Tier 4 (Terminal Clearance): AQP4 depolarization + reduced SWS → glymphatic failure → α-syn sequestration; addressable via BLT/CBT-I/sleep hygiene. The tiered architecture with built-in redundancy (BMAL1-CLOCK/NPAS2, SIRT1-SIRT3/SIRT6) explains why single-target monotherapy frequently underperforms and supports multi-tier combination approaches.

The four convergent mechanisms described above do not operate in isolation but constitute a hierarchically organized pathogenic network with defined upstream–downstream relationships and significant cross-talk. Based on the available evidence, the following hierarchical model is proposed, as illustrated in Figure 2:

(1) Upstream initiating node—REV-ERBα/NF-κB/NLRP3 neuroinflammatory axis: Dysregulation of the core clock-gene transcriptional network, particularly REV-ERBα (NR1D1), serves as the most upstream and pharmacologically tractable node. REV-ERBα simultaneously regulates dopamine biosynthesis (via TH promoter modulation), suppresses microglial NF-κB/NLRP3 inflammasome activation, and modulates complement C3/C4b-mediated synaptic phagocytosis. The availability of synthetic agonists (SR9009, STL1267) and the convergence of anti-inflammatory, neuroprotective, and DA-preserving effects make this axis the most drug-ready therapeutic target [E: direct pathway manipulation in MPTP models; P: human evidence preliminary] [40,47].

(2) Central amplifying node—NAD^+^–SIRT1 mitochondrial bioenergetic failure: Downstream of clock gene dysregulation, the decline in NAD^+^ bioavailability and SIRT1 enzymatic activity creates a metabolic amplification loop. SIRT1 deacetylation of BMAL1 and PGC-1α coordinates mitochondrial biogenesis and antioxidant defense; its impairment leads to complex I deficiency, ROS accumulation, and dopamine quinone formation—creating a bioenergetic crisis preferentially affecting dopaminergic neurons with high metabolic demand [P: robust animal model evidence; H: direct human prodromal evidence limited] [27].

(3) Effector node—C/EBPβ-dependent autophagy rhythm disruption: The NAD^+^–SIRT1 deficit propagates downstream to autophagy regulation through C/EBPβ, whose rhythmic expression is clock-dependent. Loss of C/EBPβ oscillation abolishes the transcriptional activation of autophagy effector genes (Gabarapl1, Bnip3, Ctsl), compromising chaperone-mediated autophagy (CMA)—the primary pathway for α-syn degradation [H: primarily hepatic/Drosophila evidence; extrapolation to human DA neurons cautious].

(4) Terminal clearance failure—Glymphatic impairment: The accumulation of α-syn resulting from autophagy failure is compounded by circadian disruption of glymphatic clearance. AQP4 depolarization (driven by DAPC integrity loss under oxidative stress) and reduced slow-wave sleep jointly impair nocturnal interstitial fluid turnover, completing the pathogenic cascade from clock gene dysregulation to proteinopathy [P: strong animal evidence; E: human DTI-ALPS correlational data available].

Redundancy and compensation: This hierarchical model also explains why single-target interventions may fail [48]. When BMAL1 is suppressed, CLOCK/NPAS2 heterodimers can partially compensate for transcriptional activation; when SIRT1 activity declines, the mitochondrial deacetylases SIRT3 and SIRT6 provide division-of-labor compensation for specific metabolic outputs. This functional redundancy suggests that combinatorial strategies targeting multiple nodes simultaneously (e.g., REV-ERBα agonist + NAD^+^ precursor + timed light therapy) [28] may be necessary to overcome compensatory resistance and achieve meaningful disease modification [5] (Table 2).

**Table 2:** Core Clock Gene Alterations in PD.

Gene	Physiological Function	Expression Change in PD *	Downstream Effect on Pathway	Evidence Level (E/P/H)	Model/Sample
*BMAL1*	transcriptional activation	↓	promotes α-Syn aggregation; reduces DA synthesis	P	Mouse KO + human PBMC
*CLOCK*	histone acetyltransferase activity	Mutation/↑	indirect neuronal injury	H	Human genetic association
*PER1/2*	negative feedback repression	↓	attenuated DA rhythm	P	Human PBMC + mouse model
*NR1D1*	inflammatory suppression; DA enzyme regulation	Dysregulated	NF-κB/NLRP3 activation; TH/DDC↓	E	MPTP mouse + human SN tissue
*RORα*	neuroprotective transcription	↓	loss of protective signaling	H	In vitro/inferential

* The downward arrow (↓) indicates gene downregulation.

### 4.2. Reverse Disruption of the Circadian System by PD Pathology

#### 4.2.1. “Bottom-Up” Pathological Spread in Rhythm-Related Nuclei

According to the Braak staging hypothesis, α-syn pathology involves at an early stage the dorsal motor nucleus of the vagus, locus coeruleus (LC), raphe nuclei, pontine tegmentum, and hypothalamus—key nodes in rhythm and arousal regulation. Neuropathological studies confirm a reduction in VIP-positive neurons in the SCN of PD patients [49], and lateral hypothalamic orexinergic neurons can be lost by up to 60%, closely related to daytime sleepiness [50]. This brainstem-to-hypothalamus cascade injury constitutes the anatomical basis of CRD in PD.

#### 4.2.2. Effects of Dopaminergic Input Loss on Central and Peripheral Clocks

The retina contains dopaminergic amacrine cells, whose DA release is essential for transmission of “non-image-forming light signals” by intrinsically photosensitive retinal ganglion cells (ipRGCs) to the SCN [51]. In PD patients, retinal DA levels are reduced, leading to attenuated melanopsin-mediated photoentrainment, clinically manifesting as blunted morning light response and phase delay [52]. Furthermore, striatal D1/D2 receptor activation modulates the oscillation amplitude of Per2 and Bmal1 in peripheral tissues [53]; MPTP and 6-OHDA lesion models show that disruption of the nigrostriatal pathway markedly decreases the amplitude of clock gene expression in peripheral blood mononuclear cells, consistent with patient data [54].

The microbiota–gut–brain axis exerts a significant influence on circadian dysregulation in PD [25]. The landmark study by Thaiss et al. [55] demonstrated that the host circadian clock orchestrates trans-kingdom regulation of diurnal gut microbiota oscillations through the entrainment of feeding rhythmicity: in Per1/2 double-knockout mice, the diurnal oscillations in microbial community composition and biogeographic distribution were virtually abolished, accompanied by a reduction in microbial alpha-diversity and the emergence of pronounced dysbiosis. Jet lag-induced circadian misalignment similarly precipitated gut dysbiosis, and critically, this metabolic phenotype was transmissible to germ-free (GF) recipient mice via fecal microbiota transplantation (FMT), a finding that was further corroborated in a human jet lag paradigm. With respect to PD specifically, gastrointestinal dysfunction (e.g., chronic constipation) may precede the onset of cardinal motor manifestations by years, though direct longitudinal evidence that specific microbial signatures precede motor PD remains limited and is closely correlated with gastrointestinal dysmotility—particularly chronic constipation—as well as enteric α-syn deposition within the ENS. Circadian disruption, by dismantling the temporal architecture of feeding rhythmicity and consequently perturbing diurnal oscillations in gut microbial composition [56] and metabolic output, may constitute one of the upstream driving factors of gastrointestinal pathology in prodromal PD. Furthermore, through bidirectional signaling along the gut–brain axis—encompassing vagal afferent neural pathways, microbial-derived metabolites, neuroimmune mediators, and enteroendocrine signaling—this peripheral dysbiosis may in turn compromise the stability and functional integrity of the central circadian timekeeping system, thereby establishing a self-perpetuating pathological feedback loop that reinforces both peripheral and central disease progression [57].

#### 4.2.3. Attenuation of the Pineal–Melatonin Axis

Nocturnal plasma melatonin peaks in PD patients are reduced in amplitude and phase-delayed, and significantly correlate with motor symptom scores and RBD severity [58]. Functional MRI and post-mortem studies suggest that hypothalamic gray matter atrophy and increased pineal calcification may interfere with the multi-synaptic pathway from SCN, paraventricular nucleus, superior cervical ganglion, and pineal gland, leading to reduced N-acetyltransferase (AANAT) activity and diminished melatonin synthesis [58,59]. Melatonin itself exerts antioxidant, mitochondrial-stabilizing, and α-syn oligomerization-inhibiting effects [60], and its decline further exacerbates DA neuronal vulnerability, forming a new vicious cycle.

#### 4.2.4. Iatrogenic Perturbation of Circadian Rhythms by Long-Term Levodopa Therapy

**Figure 3:**
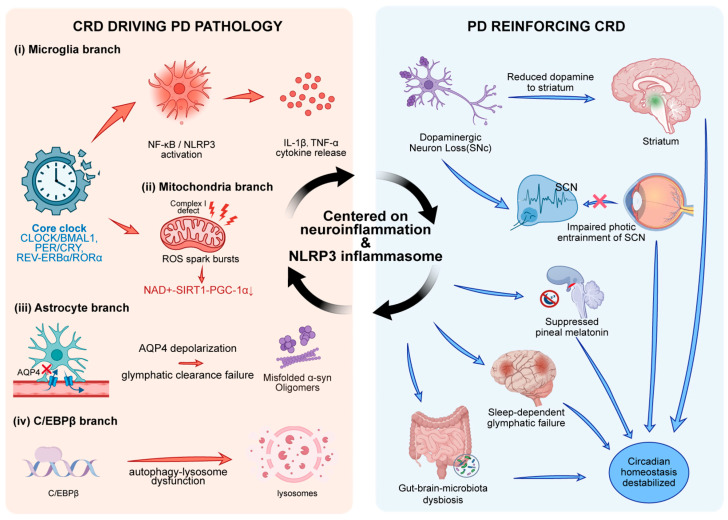
Molecular Signaling Pathway Map Underlying the Bidirectional CRD–PD Axis. Left panel: Dysregulation of the core circadian clock machinery—comprising CLOCK/BMAL1, PER/CRY, and REV-ERBα/RORα—initiates four convergent pathogenic cascades that collectively drive dopaminergic neurodegeneration: (i) microglial NF-κB/NLRP3 inflammasome activation with release of pro-inflammatory cytokines IL-1β and TNF-α; (ii) mitochondrial complex I deficiency leading to ROS overproduction secondary to suppression of the NAD^+^–SIRT1–PGC-1α axis; (iii) AQP4 depolarization causing glymphatic clearance failure and accumulation of misfolded α-syn oligomers; and (iv) C/EBPβ-mediated autophagy–lysosome dysfunction. Right panel: Progressive loss of SNc DA neurons diminishes striatal dopamine output, which impairs photic entrainment of the SCN via the RHT and suppresses pineal melatonin secretion. Concurrently, sleep-dependent glymphatic failure and gut–brain–microbiota axis dysbiosis further destabilize circadian homeostasis. The two panels complete a self-reinforcing vicious cycle centered on neuroinflammation and NLRP3 inflammasome activation.

Exogenous pulsatile DA stimulation can itself alter circadian rhythms through a well-defined neurobiological mechanism. Intermittent, pulsatile activation of striatal D1 and D2 receptors [61] by levodopa therapy cyclically induces Per2 expression via the cAMP–PKA–CREB signaling pathway [54], disrupting the phase coupling between Per2 and Bmal1 in the striatum. Long-term treatment leads to attenuation of striatal Per2/Bmal1 oscillation amplitude and progressive phase drift, constituting a direct source of peripheral molecular clock desynchronization that feeds back to the HPA axis and the pineal–melatonin axis. Clinical data confirm that PD patients on long-term levodopa therapy exhibit significantly disturbed phase relationships between cortisol and melatonin [62], and the risks of daytime sleepiness and “sleep attacks” are positively correlated with the doses of dopamine receptor agonists (pramipexole, ropinirole) [63]. This indicates that CRD in PD is the joint result of the disease itself, neurodegenerative spread, and pharmacological treatment (Figure 3).

## 5. Chronobiomarkers and Multimodal Assessment of CRD in PD

Traditional PSG-based assessment is insufficient to fully characterize CRD. With the in-depth study of chronobiomarkers in recent years, CRD assessment in PD has gradually shifted from a single sleep evaluation to a multimodal, multilevel biomarker system. This section systematically reviews recent advances in ancillary examinations and biomarkers for CRD in PD across six dimensions: objective behavioral monitoring, neuroendocrine markers, autonomic rhythm markers, molecular clock genes and peripheral metabolic markers, neuroimaging markers, and gut microbiota rhythm markers.

### 5.1. Peripheral Molecular and Metabolic Biomarkers

#### 5.1.1. Clock Gene Expression and Epigenetics

The peripheral blood expression rhythms of core clock genes such as BMAL1, CLOCK, PER1/2/3, CRY1/2, and REV-ERBα have been adopted as important tools for assessing systemic CRD. In PBMCs of PD patients, the amplitudes of BMAL1 and PER2 are reduced and their phases are disturbed, correlating with the severity of motor symptoms and sleep disturbances. This suggests that peripheral clock gene expression profiles can serve as non-invasive molecular markers of CRD in PD [64]. Hypomethylation of the NPAS2 promoter has been proposed as a potential epigenetic marker for early diagnosis of PD [5].

#### 5.1.2. The Tryptophan–Kynurenine Pathway

Tryptophan (Trp) is not only a precursor of melatonin and 5-hydroxytryptamine; its metabolites generated through the kynurenine (KYN) pathway (e.g., kynurenic acid KYNA, quinolinic acid QUIN) [65] are closely linked to neuroinflammation and neurodegeneration [66,67]. Heilman et al. found that PD patients exhibit reduced plasma Trp levels and increased KYN/Trp ratios, with quinolinic acid levels correlating with nigral pathology and motor symptom severity [68,69]. Disturbed circadian fluctuations in tryptophan pathway metabolites may simultaneously reflect rhythm dysregulation and neuroinflammatory processes, making them dual-significance candidate biomarkers.

However, the specificity of tryptophan–kynurenine metabolites as circadian disruption markers is limited [70]: these metabolites are influenced by systemic inflammation (via IDO/TDO induction), dietary tryptophan intake, renal function, depression, concomitant medication (including SSRIs and MAO inhibitors), and age. Therefore, kynurenine pathway markers should be interpreted as dual-significance indices (reflecting both rhythm dysregulation and neuroinflammatory status) rather than as specific chronobiomarkers [71].

#### 5.1.3. Other Metabolic Rhythm Markers

Circadian rhythms exist in blood glucose, insulin, lipid metabolites, and amino acid profiles, and metabolic rhythm disturbances are common in PD patients. Multi-time-point metabolomics-based sampling holds promise as a new direction for biomarker discovery in PD.

### 5.2. Neuroimaging Changes

Multimodal neuroimaging provides important support for in vivo evaluation of CRD in PD. MRI can reveal atrophy of the SCN region in the hypothalamus and weakened neuromelanin signal in the locus coeruleus; PET/SPECT imaging (e.g., ^18^F-DOPA, ^123^I-FP-CIT) demonstrates that nigrostriatal dopaminergic damage is significantly correlated with rhythm dysregulation; functional MRI can reveal circadian dynamics of the default mode network and sleep–wake-related networks. fMRI studies have shown abnormal functional connectivity in basal ganglia–thalamo–cortical networks in PD patients [72]. ^123^I-MIBG myocardial scintigraphy indicates that cardiac sympathetic denervation is closely related to autonomic rhythm abnormalities [73].

### 5.3. Objective Behavioral and Neurophysiological Monitoring

PSG is regarded as the gold standard for assessing sleep architecture and sleep-related CRD in PD patients, simultaneously recording multichannel signals such as electroencephalography (EEG), electrooculography (EOG), electromyography (EMG), electrocardiography (ECG), respiration, and oxygen saturation. In PD patients, PSG commonly shows decreased sleep efficiency, reduced slow-wave sleep (SWS), shortened REM sleep latency, REM sleep without atonia (RSWA), and increased periodic limb movements. Among these, RSWA and RBD are important prodromal warning biomarkers of PD, indicating early involvement of brainstem rhythm-regulating nuclei (e.g., locus coeruleus and pedunculopontine nucleus). PSG combined with multiscale entropy analysis of EEG can reveal early instability of NREM sleep microarchitecture in patients with PD and RBD [74,75]. Wearable actigraphy enables objective long-term recording of rest–activity rhythms [76].

By continuously recording wrist activity signals over extended periods, actigraphy can quantify multiple parameters of the rest–activity rhythm (RAR), including amplitude, acrophase, interdaily stability (IS), and intradaily variability (IV). Studies have shown that PD patients exhibit reduced RAR amplitude, decreased IS, and increased IV, with such alterations potentially appearing in the prodromal stage and serving as early screening tools. Compared with PSG, actigraphy is more suitable for long-term home monitoring and provides a realistic reflection of patients’ daily rhythm states.

EDS is a key clinical manifestation of CRD in PD, commonly assessed using the Epworth Sleepiness Scale (ESS) and the Multiple Sleep Latency Test. Pérez-Carbonell et al. noted that the pathophysiological basis of EDS involves multiple mechanisms, including hypothalamic orexin system dysfunction, dopaminergic neuronal loss, and impaired circadian regulation, and that quantitative assessment of EDS is important for both subtyping and prognostic evaluation of PD [77].

### 5.4. Gut Microbiota Rhythm Markers

A growing body of evidence confirms that the “brain–gut–microbiota axis” plays a key role in the pathogenesis and rhythm regulation of PD. Gut microbiota composition is altered in PD patients, with decreased SCFA-producing genera such as Prevotellaceae and Lachnospiraceae, and increased Enterobacteriaceae and Akkermansia. Both gut microbiota composition and their metabolites (SCFAs, bile acids, tryptophan derivatives) exhibit pronounced circadian fluctuations, the rhythmicity of which is also impaired in PD. Microbial rhythm markers (e.g., 24 h oscillations in fecal microbial abundance, circadian fluctuations in SCFA concentration) hold promise as novel biomarkers for early diagnosis and subtyping of PD [24].

**Table 3:** Multimodal Chronobiomarker Panel for CRD in PD.

Biomarker Category	Specific Indicator	Detection Method	Sample Type	Clinical Utility	Evidence Level (E/P/H)	Model/Sample
peripheral clock genes	*BMAL1/PER2* mRNA amplitude	qRT-PCR	Peripheral blood	early diagnosis; rhythm phase assessment	P	Human PBMC (PD vs. control)
neuroendocrine	Melatonin/6-SMT	ELISA; RIA	Saliva/urine	DLMO phase mapping; sleep–wake staging	E	Human saliva/urine (multi-cohort)
metabolomics	Tryptophan/kynurenine ratio	LC-MS/MS	Plasma	inflammation–rhythm crosstalk index	P	Human plasma (cross-sectional)
gut microbiota	Diurnal oscillation signatures	16S rRNA sequencing; metagenomics	Stool (timed sampling)	prodromal screening; subtype stratification	H	Human stool (pilot; limited replication)
neuroimaging	DAT-SPECT diurnal variation	Nuclear medicine	In vivo	dopaminergic reserve estimation	P	Human in vivo (small cohort)
wearable devices	IS, IV, RA	Actigraphy	Wrist-worn sensor	longitudinal ambulatory monitoring	P	Human wrist-worn (multi-cohort)
polysomnography	RBD; sleep architecture	PSG/v-PSG	Overnight recording	RBD as prodromal marker	E	Human overnight PSG (gold standard)

### 5.5. Confounders in Chronobiomarker Studies

The interpretation of chronobiomarkers in PD is complicated by multiple confounding factors that must be systematically addressed in study design and data analysis. Medication effects constitute the most pervasive confounder [78]: levodopa and dopamine receptor agonists directly alter melatonin secretion patterns and sleep architecture; MAO-B inhibitors (selegiline, rasagiline) modify monoamine metabolism with circadian-dependent pharmacokinetics; COMT inhibitors (entacapone, opicapone) prolong L-DOPA half-life and flatten dopaminergic tone oscillations [79]; anticholinergics disrupt REM architecture; antidepressants (SSRIs, SNRIs) suppress REM sleep and alter serotonin–melatonin precursor availability; hypnotics and exogenous melatonin directly mask endogenous rhythm markers; and DBS of the subthalamic nucleus can alter sleep architecture and autonomic tone [80], complicating actigraphy-derived metrics [81].

Beyond pharmacological confounders, several physiological and methodological variables must be controlled: (i) constipation and gastrointestinal dysmotility alter gut microbiota sampling reliability and drug absorption kinetics; (ii) dietary composition and meal timing entrain peripheral liver clocks and influence melatonin precursor (tryptophan) availability; (iii) sampling time-of-day standardization is critical for melatonin, cortisol, and clock gene expression measurements—single time-point samples are insufficient and multi-time-point protocols (minimum 4 time points across 24 h) are recommended; (iv) ambient light exposure history (including seasonal variation and indoor lighting conditions) modifies SCN output and retinal sensitivity; (v) disease stage and motor severity (H&Y stage, MDS-UPDRS Part III) independently affect activity rhythms and must be used as covariates.

To address these confounders, future chronobiomarker studies in PD should adopt a ‘medication-adjusted’ analytical framework: (a) record detailed medication logs including dosing times and pharmacokinetic profiles; (b) employ 48-h or longer continuous monitoring to capture intra-individual variability; (c) use linear mixed-effects models with medication type, dose, and timing as fixed effects; and (d) validate biomarkers in medication-naïve prodromal cohorts where pharmacological confounding is minimized.

## 6. Circadian-Targeted Interventions and Therapeutic Strategies for PD

### 6.1. Physical and Behavioral Interventions

Timed bright light therapy (BLT) has emerged as a promising non-pharmacological intervention for CRD in PD. Videnovic et al. conducted a randomized, placebo-controlled trial in which PD patients with excessive daytime sleepiness received bright light (1500 lux) or dim-red control light for 1 h twice daily over 14 days, demonstrating significant improvement in ESS scores and sleep quality [82]. Rutten et al. further demonstrated that BLT improves depressive symptoms in PD patients in a randomized controlled trial [83]. The therapeutic mechanism is thought to involve enhancement of SCN oscillatory amplitude via the retinal dopaminergic–ipRGC pathway; however, BLT trials in PD are heterogeneous in illumination intensity, timing, duration, and sample size, precluding definitive dose-response recommendations. Blue-enriched light (460–480 nm) may provide stronger ipRGC–SCN pathway activation [84], though caution is warranted given reduced photosensitivity in PD patients from retinal dopaminergic damage. Regular physical exercise—particularly timed aerobic exercise—can reinforce circadian rhythm amplitude [85]. Time-restricted feeding produces beneficial effects on the metabolic and nervous systems through synchronization of peripheral clocks [86]. Neuromodulation techniques such as rTMS and STN-targeted DBS exert potential improvements on sleep architecture in PD patients.

### 6.2. Pharmacological and Chronopharmacological Interventions

Exogenous melatonin (3–12 mg) has been shown to improve RBD symptoms and sleep quality in PD patients [60]. Dopaminergic medications exhibit double-edged effects: levodopa and dopamine receptor agonists can improve nocturnal bradykinesia but may induce sleep attacks and EDS. Continuous dopaminergic stimulation strategies, such as rotigotine transdermal patches or subcutaneous apomorphine pumps, are advantageous for managing nocturnal symptoms. Bedtime melatonin (3–12 mg) is effective for reducing dream-enactment behaviors and RBD severity [87]; whether it restores REM atonia per se remains less clearly established; ramelteon and agomelatine (which is also a 5-HT2C antagonist) provide dual benefits in PD with comorbid depression.

### 6.3. Potential Disease-Modifying Therapeutic Targets

SIRT1 activators (e.g., resveratrol), AMPK activators (e.g., metformin), and PPARγ agonists have shown neuroprotective effects in animal models [88]. Small-molecule agonists targeting REV-ERBα have emerged as a promising therapeutic direction within the broader framework of circadian medicine, in which pharmacological agents targeting core clock components are deliberately optimized for maximum therapeutic index through chronotherapeutic dosing strategies [48]. However, the prototype compound SR9009 has been demonstrated to exert REV-ERB-independent off-target effects, including direct modulation of cell proliferation and metabolism via non-REV-ERB pathways, necessitating cautious interpretation of its reported neuroprotective efficacy and underscoring the imperative for next-generation ligands with validated REV-ERB specificity [89]. The recent development of STL1267—a nonporphyrin-based synthetic agonist with substantially improved REV-ERB binding affinity and transcriptional specificity—represents a critical step toward resolving the selectivity limitations of first-generation compounds. Nevertheless, the clinical translation of REV-ERB-targeted therapies for PD will require the concurrent development of target engagement biomarkers (e.g., peripheral BMAL1 anti-phase transcript levels), rigorous patient stratification based on individual circadian phenotypes, and randomized controlled trials incorporating objective circadian endpoints to demonstrate disease modification rather than symptomatic benefit alone (Table 4).

### 6.4. Translational Challenges and Clinical Trial Design Considerations

The translation of circadian-targeted interventions from preclinical models to PD clinical practice faces several formidable barriers. For pharmacological agents targeting core clock components (REV-ERBα agonists, SIRT1 activators, NAD^+^ precursors), key challenges include: (i) blood–brain barrier (BBB) penetration—the prototype REV-ERBα agonist SR9009 has demonstrated limited CNS bioavailability, and next-generation ligands (e.g., STL1267) are being developed to address this limitation [89]; (ii) narrow therapeutic window—clock gene modulation exerts systemic effects, and excessive or mistimed activation may paradoxically disrupt normal circadian physiology (on-target toxicity); (iii) lack of validated target engagement biomarkers—peripheral BMAL1 anti-phase transcript levels and melatonin phase markers have been proposed but not yet validated as pharmacodynamic readouts; and (iv) all current disease-modifying evidence is preclinical (animal models and in vitro systems), and no human interventional data exist for SR9009, NAD^+^ precursors, or REV-ERBα ligands in PD specifically. The terms “preclinical” and “hypothetical” should be applied to all disease-modifying claims in this section.

For clinical trial design, several critical considerations must be addressed: (a) objective circadian endpoints—trials should incorporate dim light melatonin onset, actigraphy-derived interdaily stability (IS) and intradaily variability (IV), and multi-time-point clock gene expression profiling as primary or key secondary endpoints, rather than relying solely on subjective sleep scales; (b) stratified enrollment—iRBD cohorts offer the optimal window for neuroprotective trials, as these patients have demonstrable circadian dysfunction and a defined conversion endpoint; (c) medication confounders—trials must control for dopaminergic medication type, dose, and timing, ideally employing a wash-in/wash-out design or medication-stratified randomization; (d) combinatorial designs—given the mechanistic redundancy discussed in Section 4.1.5, 2 × 2 factorial designs (e.g., BLT × melatonin, or REV-ERBα agonist × NAD^+^ precursor) may be necessary to detect synergistic effects [90]; and (e) disease modification vs. symptomatic benefit—trials must distinguish between symptomatic improvement of sleep/circadian complaints and true disease modification (slowing of motor or cognitive progression), requiring adequately powered longitudinal designs with MDS-UPDRS and cognitive composite endpoints.

## 7. Conclusions

PD and CRD constitute a bidirectional network that forms a vicious cycle: CRD is not only a clinical manifestation of PD but also a driver of disease progression. Based on the mechanistic integration and evidence appraisal presented in this review, we propose the following testable hypotheses and practical recommendations for future research:

**H1** **(Chronobiomarker prediction).** *A combined chronobiomarker panel—comprising DLMO phase, salivary melatonin amplitude, and actigraphy-derived interdaily stability (IS)—will predict 5-year phenoconversion in iRBD patients with an AUC ≥ 0.85, significantly outperforming any single biomarker. Recommendation: A multi-center longitudinal cohort study enrolling ≥500 iRBD patients with standardized multi-time-point sampling (minimum 4 time points/24 h), 3-year follow-up, and medication-adjusted analysis is needed to validate this prediction model.*

**H2** **(Combined chronotherapy).** *Morning bright light therapy (10,000 lux, 07:00–08:00, 30 min) combined with bedtime sustained-release melatonin (3 mg) will reduce the annual rate of MDS-UPDRS Part III progression by ≥20% compared to either monotherapy in early PD patients. Recommendation: A 2 × 2 factorial randomized controlled trial (BLT × melatonin) with 18-month follow-up, objective circadian endpoints (DLMO, actigraphy IS/IV), and stratified enrollment by chronotype and disease stage (H&Y I–II).*

**H3** **(Microbiota rhythm restoration).** *Time-restricted feeding [91] (10-h eating window) combined with timed probiotic supplementation (Lactobacillus and Bifidobacterium species, administered at ZT0 equivalent) will improve PD constipation scores (measured by NMSS gastrointestinal subscale) and reduce plasma α-syn oligomer levels within 12 weeks. Recommendation: A randomized crossover trial with 12-week intervention periods, 4-week washout, 16S rRNA sequencing of timed stool samples, and plasma α-syn measurement using seed amplification assays.*

These hypotheses are grounded in the mechanistic framework presented herein and are designed to be falsifiable within feasible study designs. Their validation would establish circadian-targeted interventions as a legitimate disease-modifying strategy in PD and provide the evidence base necessary for integration into clinical practice guidelines. Precision interventions targeting circadian rhythms hold promise as a new paradigm for the prevention and treatment of PD.

## Figures and Tables

**Table 4:** Circadian-Targeted Interventional Strategies for PD.

Intervention Category	Specific Approach	Molecular/Physiological Target	Evidence Level (E/P/H)	Model/Sample
BLT	timed exposure (2500–10,000 lux; AM)	ipRGC → SCN photic entrainment	E	Human RCT (PD patients)
melatonin/agonists	exogenous melatonin; ramelteon; tasimelteon	MT1/MT2 receptors; free-radical scavenging	E	Human RCT (PD patients)
chrono pharmaco logy	optimized L-DOPA/DA agonist dosing schedule	alignment with DA synthesis circadian peak	P	Human observational + animal
exercise chronotherapy	morning aerobic exercise	BMAL1 upregulation; cortisol rhythm normalization	P	Human small RCT
behavioral/lifestyle	time-restricted feeding; sleep hygiene protocols	peripheral clock resetting; SCN–peripheral synchronization	H	Animal models; human observational

## Data Availability

No new data were created or analyzed in this study. Data sharing is not applicable to this article.

## Abbreviations

The following abbreviations are used in this manuscript:

PD	Parkinson’s disease
CRD	Circadian rhythm disruption
RBD	REM sleep behavior disorder
iRBD	Isolated RBD
EDS	Excessive daytime sleepiness
RLS	Restless legs syndrome
DLB	Dementia with Lewy bodies
PDD	Parkinson’s disease dementia
PD-MCI	PD with mild cognitive impairment
NMS	Non-motor symptoms
REM	Rapid eye movement
SWS	Slow-wave sleep
SCN	Suprachiasmatic nucleus
SNpc	Substantia nigra pars compacta
VTA	Ventral tegmental area
STN	Subthalamic nucleus
LC	Locus coeruleus
DMN	Default mode network
DA	Dopamine
TH	Tyrosine hydroxylase
α-syn	α-synuclein
AQP4	Aquaporin-4
DAPC	Dystrophin-associated protein complex
BMAL1	Brain and muscle ARNT-like 1
CLOCK	Circadian locomotor output cycles kaput
PER2	Period circadian regulator 2
NR1D1	Nuclear receptor subfamily 1 group D member 1
NURR1	Nuclear receptor related 1
NR4A2	Nuclear receptor subfamily 4 group A member 2
NAD^+^	Nicotinamide adenine dinucleotide
SIRT1	Sirtuin 1
SIRT3	Sirtuin 3
SIRT6	Sirtuin 6
PGC-1α	PPARγ coactivator 1-alpha
NAMPT	Nicotinamide phosphoribosyl transferase
NMN	Nicotinamide mononucleotide
NR	Nicotinamide riboside
PINK1	PTEN-induced kinase 1
NLRP3	NLR family pyrin domain containing 3
NF-κB	Nuclear factor kappa B
IL-1β	Interleukin-1 beta
IL-6	Interleukin-6
TNF-α	Tumor necrosis factor alpha
TLR4	Toll-like receptor 4
C3/C4b	Complement component 3/4b
C/EBPβ	CCAAT/enhancer-binding protein beta
ULK1	Unc-51-like autophagy-activating kinase 1
LC3B	Microtubule-associated protein 1 light chain 3 beta
TFEB	Transcription factor EB
ROS	Reactive oxygen species
DAQ	Dopamine quinones
AANAT	N-acetyltransferase
VIP	Vasoactive intestinal peptide
CMA	Chaperone-mediated autophagy
TTFL	Transcription–translation feedback loop
RHT	Retinohypothalamic tract
ipRGC	Intrinsically photosensitive retinal ganglion cells
HPA	Hypothalamic–pituitary–adrenal
BBB	Blood–brain barrier
FMT	Fecal microbiota transplantation
SCFA	Short-chain fatty acid
CSF	Cerebrospinal fluid
MPTP	1-methyl-4-phenyl-1,2,3,6-tetrahydropyridine
MPP^+^	1-methyl-4-phenylpyridinium
ZT	Zeitgeber time
ESS	Epworth Sleepiness Scale
